# Comparative genetic analysis of frequentist and Bayesian approach for reproduction, production and life time traits showing favourable association of age at first calving in Tharparkar cattle

**DOI:** 10.5713/ab.23.0025

**Published:** 2023-06-26

**Authors:** Nistha Yadav, Sabyasachi Mukherjee, Anupama Mukherjee

**Affiliations:** 1 Animal Genetics and Breeding Division, ICAR-NDRI, Karnal, Haryana-132001, India

**Keywords:** Bayesian Approach, Lifetime Traits, Least Squares Maximum Likelihood Method (LSML), Production, Reproduction, Tharparkar

## Abstract

**Objective:**

The present study was aimed primarily for estimating various genetic parameters (heritability, genetic correlations) of reproduction (age at first calving [AFC], first service period [FSP]); production (first lactation milk, solid-not fat, and fat yield) and lifetime traits (lifetime milk yield, productive life [PL], herd life [HL]) in Tharparkar cattle to check the association of reproduction traits with lifetime traits through two different methods (Frequentist and Bayesian) for comparative purpose.

**Methods:**

Animal breeding data of Tharparkar cattle (n = 964) collected from Livestock farm unit of ICAR-NDRI Karnal for the period 1990 through 2019 were analyzed using a Frequentist least squares maximum likelihood method (LSML; Harvey, 1990) and a multi-trait Bayesian-Gibbs sampler approach (MTGSAM) for genetic correlations estimation of all the traits. Estimated breeding values of sires was obtained by BLUP and Bayesian analysis for the production traits.

**Results:**

Heritability estimates of most of the traits were medium to high with the LSML (0.20±0.44 to 0.49±0.71) and Bayesian approach (0.24±0.009 to 0.61±0.017), respectively. However, more reliable estimates were obtained using the Bayesian technique. A higher heritability estimate was obtained for AFC (0.61±0.017) followed by first lactation fat yield, first lactation solid-not fat yield, FSP, first lactation milk yield (FLMY), PL (0.60±0.013, 0.60±0.006, 0.57±0.024, 0.57±0.020, 0.42±0.025); while a lower estimate for HL (0.38±0.034) by MTGSAM approach. Genetic and phenotypic correlations were negative for AFC-PL, AFC-HL, FSP-PL, and FSP-HL (−0.59±0.19, −0.59±0.24, −0.38±0.101 and −0.34±0.076) by the multi-trait Bayesian analysis.

**Conclusion:**

Breed and traits of economic importance are important for selection decisions to ensure genetic gain in cattle breeding programs. Favourable genetic and phenotypic correlations of AFC with production and lifetime traits compared to that of FSP indicated better scope of AFC for indirect selection of life-time traits at an early age. This also indicated that the present Tharparkar cattle herd had sufficient genetic diversity through the selection of AFC for the improvement of first lactation production and lifetime traits.

## INTRODUCTION

Tharparkar, the lyre-horned dual-purpose zebu cattle with its origin in the Tharparkar district of the Sindh province, Pakistan is currently one of the most well-established cattle in Indian and the world. The breed is more famed as a dairy animal owing to its desirable attributes of high production capacity and climate resilience under hot arid unfavorable environmental conditions [1]. The breed has been found to maintain a higher level of production concomitant with low reduction in feed intake, growth rate, and reproductive functions even in conditions of heat stress [2], harsh climate, scarce feed, and fodder inputs, among others. Studies based on phenotypic performance of reproductive traits with production potential of Tharparkar cows and expression of reproductive markers viz. anti-Mullerian hormone are important to enhance future production and significant for selection in any breeding program of zebu cattle [3]. However, most of the economically important traits are still unexplored in this breed while comparing the precision of estimates with traditional and Bayesian methods.

Economic dairy production rests upon superior production and efficient reproduction of the herd along with sufficient diversity. The information on genetic parameters like heritability, repeatability and genetic correlation is a prerequisite for making efficient selection strategies. Desirable selection in livestock can be done by either prediction equations or by considering association studies of early expressed traits viz. age at first calving (AFC), first service period (FSP), first dry period, first calving interval which affects production in long term viz. First lactation milk yield (FLMY), peak milk yield, lactation length (LL), dry period, herd life (HL), productive life (PL), breeding efficiency, and lifetime milk yield (LTMY). The economics of these dairy traits are directly affected by reproduction and herd survival as significant association was observed in different cattle and buffalo breeds [4–6]. A combined study including phenotypic records of growth from birth to regular interval and livability with production till disposal in herd gives a direction to genetic improvement on phenotypic scale [7,8].

Most of the economic traits in dairy animals are quantitative in nature and follows non-Gaussian distribution with continuous values having wide range of variability determined by the genetic makeup of the individuals and the environment in which they are reared. As such maximal production is achieved when animals are sound in early expressed traits of the fertility cycle. Consequently, reproductive management enables improvement in herd performance in terms of production (milk yield, composition, and stayability) and functional (reproduction, growth, digestion, and ailments issues) traits. Progress in future fertility and long-term survival of high-producing cows aids in balanced selection and culling decision-making systems [9]. Hence, it is imperative to combine reproductive management strategies with new technologies that are user-friendly and employs versatile models to simulate production ability for consultation in dairy herds [10].

Sample size is a prime consideration in conducting any statistical analysis. The Bayesian approach has no such prior assumption about sample size as it assures higher coverage, more powerful estimates and robust results even in small sample populations [11]. It was reported that when the sample size is equal or larger than the number of parameters either Frequentist or Bayesian estimation can be applicable. However, for limited sampling Bayesian approach is more reliable alternative which provides posterior distribution, credible intervals, convergence diagnostics and graphical visualization for validation [12]. The Markov chain Monte Carlo (MCMC) method of Gibbs sampling (GS) is a particular Bayesian approach increasingly used in animal breeding to estimate model parameters by generating random variables from full conditional posterior distributions. This approach offers a more natural marginalization process based on having an accurate prior probability to deal with nuisance parameters [13].

Efficient selection strategies used by breeders are based on variability of data distribution for getting improvement in desired characters. The application of advance methods of genetic analysis provides a crucial tool for improving the production traits in indigenous milch breeds and helps economize the herd productivity. Quantitative analysis for viable traits of these economically important breeds is a complex phenomenon owing to continuous scale on phenotypic as well as genotypic line [14]. The likelihood-based methods, such as maximum likelihood (ML), restricted maximum likelihood (REML), least square maximum likelihood (LSML) and linear regression techniques are conventionally used for animal breeding data analysis, where outcome simply equals the linear predictor [15]. These methods are useful and efficient when samples have linearity and normal distribution. However, the approximations like assumption of normal distribution, defined confidence intervals are not found in the sample population most of the time; moreover, these methods lack accuracy and require huge memory space. The Bayesian estimate provides a better alternative to overcome these issues by employing probability intervals for estimation of genetic parameters. Application of Bayesian approach to solve problems in breeding is a novel idea which provides a general, coherent methodology with inbuilt self-evident system. Bayesian paradigm introduced knowledge of priori distribution about parameters of interest for getting precise estimates of posterior probabilities and credible interval regions (95% probability in a fixed interval) for reliable parameters by various convergences diagnostic [16]. While confidence interval of the classical approach in similar observations introduces more errors in parameter estimation [17]. The Bayesian methods also enable estimation within a single bivariate analysis while providing flexibility to define model for each trait with homogenous or heterogeneous contemporaries, which is not possible with the likelihood methods. The improvement of ability to interpret uncertainty in sequential estimation of posterior distributions is perhaps the greatest benefit of Bayesian methods [18].

It is important to know the effectiveness of Bayesian approach vis-a-vis other methods for genetic evaluation and ranking of sires based on the 1st lactation traits. To our knowledge, no studies have been carried out in the Tharparkar cattle for comparative genetic evaluation using the frequentist LSML approach and more advanced Bayesian multi-trait Gibbs sampling animal model (MTGSAM). This comparison between these two methods (conventional v/s advance) to check their efficacy with a finite sample size of this Indigenous milk breed was one of the purposes of the present study. Bivariate analysis for the estimation of genetic parameters has not been attempted with heterogeneous contemporary groups of affecting factors for AFC and FSP in the Tharparkar breed. Therefore, present study was planned to obtain comparative analytical evidence using Frequentist and Bayesian approaches while considering reproduction, production and lifetime traits as selection criteria and to estimate the breeding values of sires for milk production and composition traits in Tharparkar cattle.

## MATERIALS AND METHODS

### Data structure

All the reproduction, milk parameters (production and composition) and lifetime traits were generated from history-cum-pedigree sheet and production records of 964 Tharparkar cows over a period of 30 years (1990 through 2019), maintained at ICAR-NDRI Karnal, Haryana. Data optimization was performed by normalization and standardization methods after compilation, editing and discarding the suboptimal records. Outlier animals from the standardizing criteria *viz*. LL less than average days (<100 days), milk yield less than average yield (<500 kg) and fat percent less than standard (<3.5%) were expelled for final analyzable samples in our study. Non-genetic factors such as period, season of calving/birth and AFC as covariate were classified into different fixed sub-classes and animal/sire was considered as random genetic factor to assess the effect on the considered traits in the present study. The year of calving/birth had been classified into four seasons e.g. summer (April to June), rainy (July to September), autumn (October to November) and winter (December to March) based on recorded and analyzed meteorological factors at CSSRI, Karnal, India for temperature fluctuations, relative humidity, prevalent geo-climatic conditions. The region is at an altitude of 235 to 252 meters from the sea level, latitude 29.43°N and longitude 77.2°E with 10°C to 45°C temperature range.

Whole data had been classified into six and seven periods by taking five years in a group based on the year of calving (1990 through 2019) and year of birth (1986 through 2019), respectively. AFC was taken as covariate for all traits except for AFC as a continuous trait. Covariate AFC has been classified in eleven classes based on formula given by Sturges [19] as:


$$Number\;of\;age\;group=\frac{Range}{1+3.322{log}_{10}N}$$

Where, N = No. of observations; range = maximum – minimum

### Considered traits

Reproduction traits include calving and service parameters which affect per animal production and survival in herd. Production traits include traits of economic importance such as milk production and composition traits which have direct effect on profitability of dairy sector as well as individual farmer’s income and livelihood. Lifetime traits include PL span of animals which reflect survival and longevity in herd.

*Reproduction traits*:

- Age at first calving
- First service period

*Production traits*:

- First lactation 305 days / Less milk yield (FL305MY)
- Solid-not fat yield
- Fat yield

*Lifetime traits*:

- Lifetime milk yield
- Productive life
- Herd life

### Statistical analysis

Descriptive statistics of considered quantitative traits were estimated by using R software (version 4.2.0) [20]. Data has been statistically analyzed by using LSML and Bayesian approach by using suitable software packages such as Harvey [21] and BLUPF90 family [22], respectively. Sire evaluation taking the production traits was also carried out after estimation of breeding value by both the methods using Model 8 under Harvey [21] and BLUP animal model under BLUPF90 package.

#### LSML approach

The effects of genetic (sire) and non-genetic factors (periods, season of birth/calving and AFC) were assessed by LSML method [21].

#### Bayesian approach

The (co)variance component and genetic parameters viz. correlations and heritability estimates were calculated in terms of posterior densities by using multiple iterative cycles of Markov chains for GS. This Bayes theorem has given joint probability of priori and likelihood values from data distribution. More uniform environmental conditions increase the heritability and vice-versa. Data were subjected to analysis by using software packages among the list of BLUPF90 family. RENUMF90 software was a preliminary in this series which generated a renumbered file by removing alpha-numeric values. Standardized (Gaussian distribution) Gibbs samples from GIBBS2F90 software gives binary results. POSTGIBBSF90 package was used to read these binary results and present them in a readable format. Breeding Values were estimated by using BLUPF90 software. Results were based on various standardizing criteria such as Monte Carlo error (MCE) for standard deviation (SD) for accuracy, constant trace plot and Histogram. Proper standardization of Gibbs sampler can be ensured by visualization of stable and normalized marginal posteriori [23]. The final POSTGIBBSF90 estimates by the Bayesian approach with a multi-trait animal model used the following mixed model equation:


Y=Xβ+Zμ+e

Where,

Y = (n×1) Vector of observed dependent variables, viz. reproduction, production and lifetime traits
X = Incidence matrix (vector of 1’s) relating the fixed effects to the individuals
β = (p×1) Vector of fixed effects (year, season)
Z = Incidence matrix relating the random effects to each individual
μ = (q×1) Vector of random effects (all animals in the pedigree file)
e = Random error, normally distributed (mean = 0, residual variance = σ^2^_e_)

Following models were used for various reproduction, production, and lifetime traits:

*For FSP, FLMY, FLFY, FLSNFY, LTMY, PL, and HL*:


$${\text{Y}}_{\text{ijklm}}=\mu +{\text{P}}_{\text{i}}+{\text{Sn}}_{\text{j}}+{\text{Ag}}_{\text{k}}(\text{cov})+{\text{S}}_{1}+{\text{e}}_{\text{ijklm}}$$

Where,

Y_ijklm_ = Observation of mth animal having lth sire effect, kth age group at first calving, calved in jth season, ith period
μ = Overall mean
P_i_ = Effect of ith period of calving (i = 1 to 6)
Sn_j_ = Effect of jth season of calving (j = 1,2,3,4)
Ag_k_(cov) = Effect of kth age group in first calving (k = 1 to 7), AFC will be taken as covariate
S_l_ = Effect of lth sire (l = 8)
e_ijklm_ = Random error, N (0, σ^2^_e_)

*For AFC:*


$${\text{Y}}_{\text{ilkl}}=\mu +{\text{P}}_{\text{i}}+{\text{Sn}}_{\text{j}}+{\text{S}}_{\text{k}}+{\text{e}}_{\text{ijkl}}$$

Where,

Y_ijkl_ = Observation of lth animal calved in ith period, jth season, kth sire
μ = Overall mean
P_i_ = Effect of ith period of birth (i = 1 to 7)
Sn_j_ = Effect of jth season of birth (j = 1,2,3,4)
S_k_ = Effect of kth sire
e_ijkl_ = Random error, N (0, σ^2^_e_)

#### Standardization of Gibbs sampler

The Gibbs sampler was used to generate random samples from Bayes theorem with conditional joint probability by successive sampling resulted in marginal posterior distribution. Estimates generated in several standardized parameters such as iterative, burn in and thinning cycles were compared to get the representative samples for the desired convergence. First non-converging samples were discarded as burn-in. Burn-in and cycles of thinning intervals in each sampling round generate final effective samples for considered traits. MTGSAM approach was considered to analyze genetic and phenotypic correlation between all reproduction, production and lifetime traits in Tharparkar. For this a total of 2,900 Gibbs samples were finalized to get standardized sampling parameters after various trials while each 10th cycles to be stored after discarding initial 1,000 cycles from overall sample chain of 30,000 for all considered traits in Tharparkar.

## RESULTS

### Descriptive statistic

The descriptive statistics viz. mean±standard error (SE), coefficient of variation, SD, and minimum, maximum ranges was estimated to generate a summary of the present data set (Table 1). These initial mean values can be used as priori distribution in Bayesian statistics for considered data distribution in single or multi-trait analysis [24]. Coefficient values ranged between 14.62 (AFC) and 63.50 (FSP), which indicated less and more variability for respective traits in the sampled Tharparkar population (Table 1).

### Least-square mean and affecting factors

Least-square Means and effect of various genetic and non-genetic factors were estimated by the LSML (Harvey [21]) approach for Tharparkar cattle (Table 2). The average AFC in our herd was found as 1,093.74±31.83 days. Season has significant effect at p<0.01 while other nongenetic and genetic factors were found to be non-significant. This might be due to differences in feeding, managemental practice and environmental condition in different seasons. Average FSP, FLMY, first lactation solid not fat yield, first lactation fat yield, LTMY, production life (PL) and HL were found as 152.49±17.25 days, 1,777.95±211.60 kg, 2,962.03±186.61 kg, 1,633.76± 105.86 kg, 7,875.34±441.11 kg, 5,373.92±94.63 days and 6,665.18±92.84 days respectively (Table 2). All non-genetic and genetic factors were found to be non-significant for FSP, FLMY, and FLSNFY. This indicates that these traits are not affected by considered factors or might be due to small sampling structure. Period of calving has significant effect for FLFY (p<0.01) and LTMY (p<0.05) and other non-genetic factors were found to be non-significant while genetic factor was only significant for later trait. All non-genetic and genetic factors were found to be non-significant for PL and HL in this data set. Covariate AFC had non-significant effect on all considered traits.

### Bayesian posterior densities and genetic evaluation

**Figure 1:**
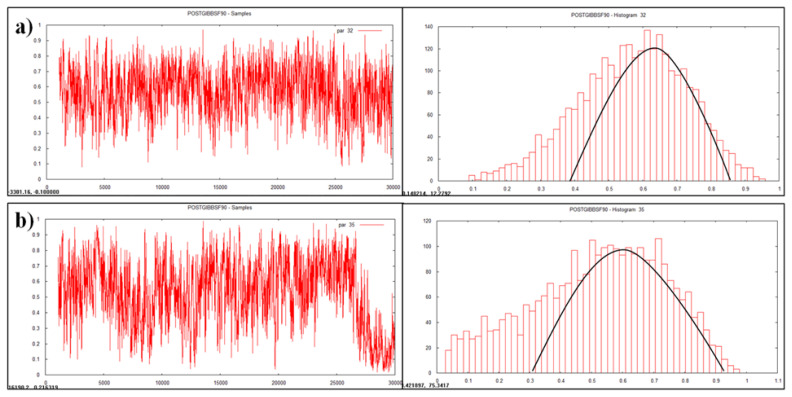
Plotting of heritability estimate by trace plot and histogram (a) age at first calving, (b) first service period.

**Figure 2:**
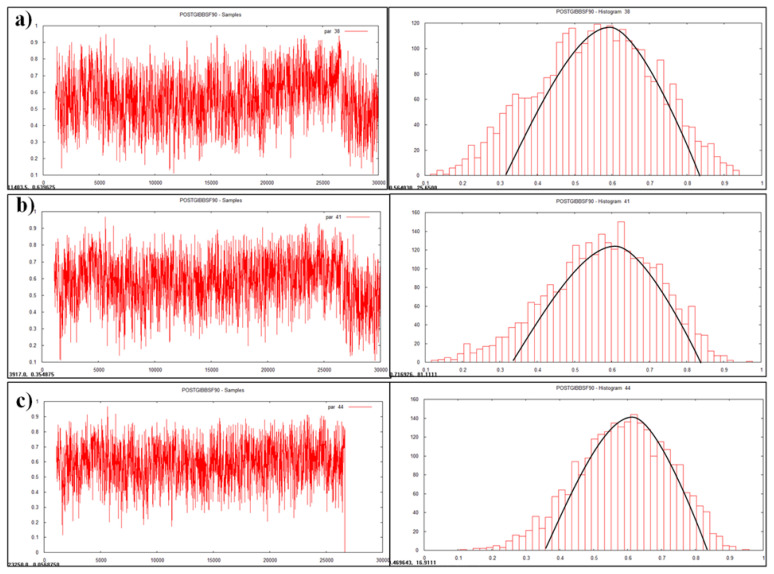
Plotting of heritability estimate by trace plot and histogram (a) first lactation milk yield, (b) first lactation solid-not fat (SNF) yield, (c) first lactation fat yield.

**Figure 3:**
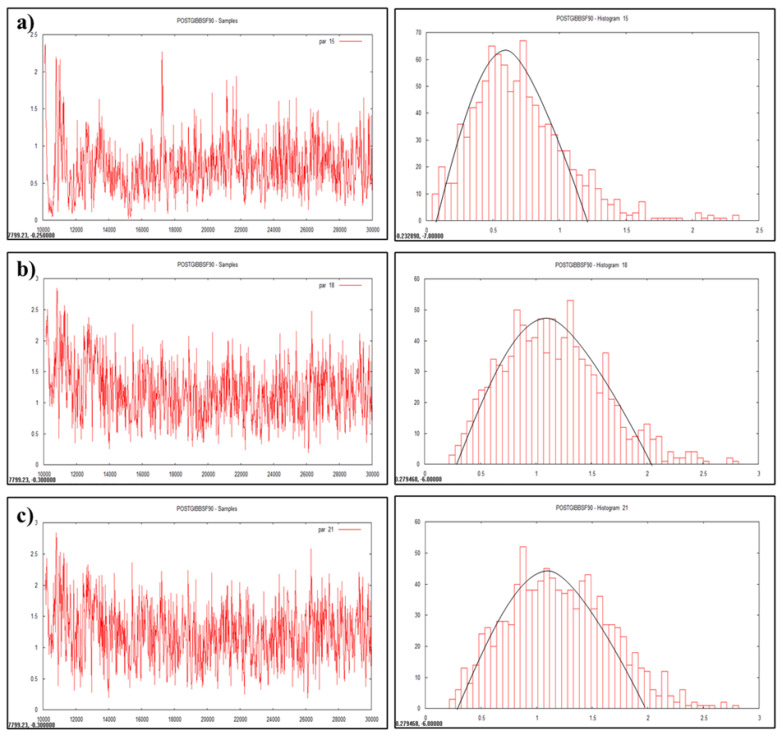
Plotting of heritability estimate by trace plot and histogram (a) lifetime milk yield, (b) productive life, (c) herd life.

The results for variance component and heritability estimates obtained through GS are presented in Table 3 and Figure 1, 2 and 3. Results were subjected for comparison with conventional method (LSML v/s Bayesian) to generalize this application in animal breeding (Table 4).

Higher variances and variability was noticed for LTMY among all considered economic traits in the present work (Table 3). Highest phenotypic variance of LTMY (12,187,000) was noticed followed by comparative lower values for HL and PL (660,240 and 654,990) subsequently. Overall genetic variance of HL was the lowest among lifetime traits. Highest phenotypic variability and wide range (3,791,300±175,930) was noticed for LTMY followed by environmental and genetic deviations (3,157,800±112,830 and 2,201,100±152,470). Less variability was noticed for genetic deviation of HL (118,870±15,297) among lifetime traits. Genetic variability was higher for HL as compared to PL instead of higher phenotypic variability of HL.

Higher phenotypic variance was noticed for all considered traits among sources of variances for Tharparkar cattle (Table 3). The total phenotypic variances (35,734) among reproduction traits were higher for AFC followed by genetic and environmental variances (21,689 and 14,045). The estimates of variability and range of posterior densities among reproduction traits were higher and wide for phenotypic variation of FSP (10,856±1,448.4) followed by genetic and environmental variation (8,456.1±1,192.2 and 4,975.8±406.44).

Variance and variability components were higher for production traits as compared to reproduction traits in present study (Table 3). Higher phenotypic variance was noticed for FLMY (4,453,900) among all variances of production traits followed by genetic and environmental variances (2,589,900 and 1,864,000). Followed by positive phenotypic variances (4,702,600 and 1,345,100), genetic (2,831,800 and 811,450) and environmental (1,870,800 and 533,640) variances of FLFY and FLSNFY. Higher variability was noticed for phenotypic variation of FLMY (2,617,600±408,740) subsequently for FLFY and FLSNFY (2,125,000±211,240 and 577,850± 64,678).

Marginal posterior mean for variance component and heritability are more informative. Heritability estimates were found as 0.61±0.017 and 0.57±0.024 for AFC and FSP; 0.57± 0.020, 0.60±0.013, and 0.60±0.006 for FLMY, first lactation solid-not fat yield and first Lactation Fat yield; 0.24±0.009, 0.42±0.025, and 0.38±0.034 for LTMY, PL, and HL respectively (Tables 3 and 4).

#### Credible interval

Highest phenotypic, genetic, and environmental variance and variability was noticed for LTMY, while Lowest for FSP from our study (Table 3). The credible intervals or highest posterior density (HPD) region was depicted as lower and higher range and considered as a measure of reliability, described as the interval range which includes 95% of samples for all estimates of (co)variance components and genetic parameters (Table 3). Bayesian analyses for marginal posterior densities of the genetic parameters with accuracy by visualizing the errors [25].

#### Effective iterative trials

The number of effective samples varied from 50 to 233, 35 to 571 and 19.5 to 783 for reproduction, production and lifetime traits respectively (Table 3). Sufficient estimates for all measures of central tendency and the HPD region at 95% credible interval for each parameter were obtained with these generated iterative sample sizes.

#### Geweke diagnostic

Geweke diagnostic values were ranging between −0.30 to 0.30 for the traits under consideration in the present study (reproduction traits: −0.30 to 0.17, production traits: −0.24 to −0.10, lifetime traits: −0.21 to 0.30) in Tharparkar cattle (Table 3). Geweke’s diagnostics were in a narrow range (near one) for production traits, followed by the lifetime and reproduction traits, which indicated high to low convergence, respectively.

### Visualization by trace plot and histogram

Data visualization is another advantageous feature of Bayesian approach which enables accuracy, automation, and cross validation of statistical analysis. Statistically analyzed genetic parameters were visualized and validated by trace plot and histogram of the heritability estimate (Figures 1, 2, and 3).

### Comparative heritability estimate from Harvey and Bayesian methods

The heritability estimates using the LSML and Bayesian approach were summarized to compare accuracy (Table 4). The heritability values ranged from medium to higher (0.20± 0.44 to 0.49±0.71 and 0.24±0.009 to 0.61±0.017) by the LSML and Bayesian analysis, respectively (Table 4).

Heritability estimates were higher ranging between 0.24±0.009 to 0.61±0.017 by the MTGSAM analysis (Tables 3, 4, and 6). The highest heritability was noticed for the AFC (0.61±0.017) followed by FLFY, FLSNFY, FSP, FLMY, PL, HL (0.60±0.013, 0.60±0.006, 0.57±0.024, 0.57±0.020, 0.42± 0.025, 0.38±0.034) and the lowest for the LTMY (0.24±0.009) in Tharparkar cattle.

### Correlation component between reproduction, production and lifetime traits

Genetic and phenotypic correlations estimated using the LSML technique wss presented in Table 5. Negative (−0.0037 to −0.3821) and positive (0.0330 to 0.9622) correlations were obtained for various trait combinations (Table 5). Positive genetic and phenotypic correlations were high for FLFY-FLSNFY followed by high to moderate for FLMY-FLFY, FLMY-FLSNFY; slightly higher for FSP-FLSNFY, FSP-FLFY; low for FSP-FLMY FSP-AFC and lowest for AFC-PL in our study. Negative genetic and phenotypic correlations were moderate for FSP-LTMY, FSP-PL, AFC-FLFY, PL-FLSNFY, PL-FLFY; low for AFC-FLSNFY, AFC-FLMY, AFC-LTMY, PL-FLMY, LTMY-FLFY and the least for LTMY-FLSNFY in our study.

Genetic and phenotypic correlations were estimated using Gibbs Sampling with multi-trait Animal model (MTGSAM) from Bayesian analysis (Table 6). These results were useful for the interpretation and genetic improvement of the correlated traits of reproductive and productive importance by indirect selection in Tharparkar cattle. Correlation component was negative for AFC (moderate to high: −0.33±0.029 to −0.59±0.24) and FSP (low to moderate: −0.04±0.041 to −0.38±0.101) with lifetime traits (PL and HL), while other traits were positively correlated in this study (Table 6). High genetic correlation was observed for FLFY-FLSNFY (0.99± 0.0005) followed by high to moderate for PL-HL, FLMY-FLSNFY, FLMY-FLFY, AFC-FLSNFY, AFC-FLFY (0.95± 0.0032, 0.93±0.007, 0.92±0.007, 0.85±0.007, 0.80±0.008); medium for AFC-FLMY, FSP-FLFY, FSP-FLSNFY, FSP-FLMY (0.68±0.018, 0.65±0.074, 0.63±0.075, 0.59±0.056); slightly higher for FLSNFY-PL, FLFY-PL, AFC-FSP, FLSNFY-HL, FLFY-HL, FLMY-HL (0.35±0.091, 0.33±0.093, 0.32± 0.050, 0.26±0.121, 0.25±0.120, 0.23±0.117) and the least for FLMY-PL (0.13±0.133), while it was negative for AFC-PL, AFC-HL, FSP-PL and FSP-HL (−0.59±0.19, −0.59±0.24, −0.38±0.101 and −0.34±0.076) in our study. Phenotypic correlation was high for FLSNFY-FLFY (0.99±0.0003), followed by high to moderate for HL-PL, FLSNFY-FLMY, FLFY-FLMY, FLSNFY-AFC, FLFY-AFC, FLMY-AFC (0.96±0.0032, 0.93± 0.006, 0.92±0.006, 0.87±0.004, 0.85±0.005, 0.77±0.013); medium for FLSNFY-FSP, FLFY-FSP, FLMY-FSP, FSP-AFC (0.64±0.037, 0.64±0.036, 0.54±0.049, 0.46±0.037); slightly higher for FLMY-HL, FLSNFY-PL, PL-FLFY, HL-FLFY, HL-FLSNFY (0.25±0.047, 0.24±0.0553, 0.23±0.0691, 0.23±0.0525, 0.23±0.0488) and the least for PL-FLMY combine (0.19± 0.054), while it was negative for AFC-PL, AFC-HL, FSP-PL and FSP-HL (−0.33±0.029, −0.21±0.029, −0.16±0.067 and −0.04±0.041), respectively in the present study.

### Estimation of breeding value

To ensure the dissemination of superior germplasm from elite bulls estimated breeding values (EBVs) were calculated for sires by using both approaches; the Frequentist least squares method and Bayesian application using animal model. Sires having more than three progenies were considered in this study. The EBVs of Tharparkar sires for production traits were interpreted for effectiveness of sire evaluation (Table 7).

## DISCUSSION

### Inference about least squares mean and various factors included in the models

AFC of the Tharparkar cattle in our study was less, indicating the animals of ICAR-NDRI herd had a better reproductive potential, compared to AFC estimates of Tharparkar cattle reported earlier 1,876.17±40.66 [26], 1,821±37 [27], and 1,769.07±29.80 [28]. The effects of the period and season of birth on the AFC in the Tharparkar was similar to an earlier report [26]. The estimated value of FSP of Tharparkar cattle in our study resembled previous reports 152.04±4.58 [26], 132±9 [29], and 151±11 days [30], respectively, and differed with the result of Mishra et al [28] with 117.53±2.39 days. The effect of period of calving was not in agreement with previous research [26,29,30] as they found it significant on FSP. Average FLMY in the Tharparkar was less in the present study than that of reported earlier (1,822.65±70.2 kg) [31]; however, higher than that of reported at 1,019±20 kg [32]; and similar to another report with 1,618±70 kg [33]. While the effect of season on FLMY was similar to our study, the effect of period on FLMY was unlike the previous results [32,33]. The estimates and the factors affecting the LTMY in the Tharparkar cattle in this study was also similar to earlier findings with 8,013.07±322.08 kg [34]. The estimates for PL and HL in Tharparkar cattle were found to be in proximity with the results of Sharma and Singh [35] with 1,460.00 and 2,657.2 days, respectively; however, were different with the findings of Gahlot [36] with 1,867.34±96.82 and 3,540.57± 29.74 days, respectively. The estimate of HL in the Tharparkar cattle was higher compared to earlier reports of Kumar [34], Pirzada [37], and Choudhary et al [38]) with 3,240±172, 2,884.86±49.67 and 3,080.55 days respectively. All the genetic and the non-genetic factors had a similar influence on the PL and HL in Tharparkar cattle as reported by Sharma and Singh, and Pirzada [35,37]. These results indicated that the NDRI Tharparkar herd is thriving well in terms of production and survival capacity presently and has the possibility of continuing under changing climatic conditions.

### Variance components

In the present study, more informativeness of the posterior estimates from the mixed model multi-trait Bayesian analysis technique explains the 95% HPD, SD, and convergence criteria in the Tharparkar (Tables 3 and 6). Our results revealed the marginalization of data by converging them to a point estimate by generated effective samples of distinct iterations utilizing the Bayesian technique, which was similar to bootstrapping phenomenon that leads to normalization even with finite or small sample size [39].

Moderate to higher genetic variance as compared to environmental variance for AFC and FSP indicated lesser environmental effect, therefore, having more scope for direct selection in this Tharparkar herd for improving the reproductive potential. This can be stabilized towards the desired direction as lesser AFC and FSP are profitable for the herd. There is also further scope of selection and improvement for production traits as well due to higher variability as compared to reproduction traits. A more disperse pattern of variables for FLMY signified a less stabilized population, indicating further scope for selection and genetic improvement with this traits, while lesser scope for selection for the FLSNFY due to lesser variance and variability. LTMY in the Tharparkar cattle had a higher selection scope due to wide variability. Among the lifetime traits, the HL had a more diverse range in genetic selection which indicated further scope for directional selection in this Tharparkar herd. Even if the sample size was small, higher estimates of the posterior distribution in this study were obtained, implying further scope of genetic improvement in Tharparkar cattle.

### Posteriori of heritability estimates

Heritability is a dynamic trait as it varies from population to population and from environment to environment. Heritability value of various traits was estimated and visualized as graphs (Figures 1, 2, and 3) in the multivariate analysis through the Bayesian approach (Table 3) in the Tharparkar cattle. Good convergence was indicated by the constant trace plot and a bell-shaped histogram indicated normalization of the data till desired distribution was obtained to get the point estimates even more constant and symmetrical for production traits as compared to reproduction and lifetime traits (Figures 1, 2, and 3).

Heritability estimates for the reproduction and production traits were low to moderate by Bayesian approach (Table 3) in the Tharparkar cattle. Higher heritability estimate of AFC indicated higher genetic variance and variability for AFC among other reproduction traits. Comparatively higher heritability for all the production traits (FLMY, FLSNFY, and FLFY) indicated higher genetic variance than environmental variance, which was desirable. Slightly higher to moderate heritability estimates were obtained by both the statistical approaches for lifetime traits in the Tharparkar cattle.

### Convergence diagnostic

Convergence diagnostics for Gibbs samplers-MCMC was indicated with the graphical plotting (Figures 1, 2, and 3) and the posterior densities (Monte Carlo Error and Geweke values) of estimated parameters (Table 3) in this study. Geweke statistics is a diagnostic tool to tackle convergence problems by knowing the effective sample size and to determine when it is safe to stop sampling. Likewise, after performing lots of iterative trails the final genetic parameters had very low Geweke’s diagnostic values (Table 3). Equality testing of two equal means at first 10% and last 50% part in Markov chain indicates that the samples are drawn from the stationary distribution of the chain [40]. This is useful in comparing within-chain and between-chain variances analogous to a classical analysis of variance.

### Comparative heritability estimates

Different heritability estimates can be explained by flexibility and cross validation using graphs in the Bayesian approach; however, no such freedom/tool is available in the LSML approach. Interaction of traits is an important factor in the multi-trait analysis. Moderate to high heritability estimate in a narrow range by multi trait analysis explain direct or indirect influence for considered traits. In general, correlation or regression estimate is more precise if the relatives have close relationship and less precise with small sample size [12].

The Monte Carlo error is the error in parameter estimation with defined number of samples used from the Gibbs chain and is inversely proportional to the length of the Gibbs chain [23]. These indicate sufficient numbers of effective Gibbs samples to give reliable estimates. Comparative heritability estimates through the LSML and Bayesian techniques (Table 4) also indicated accuracy and precision with the later by giving estimates with low error *i.e*. MCE (Tables 3 and 6), even with a small sample size in comparison to the LSML approach (Tables 4 and 5). The SE was comparatively higher in the LSML approach ranging between 0.441–0.731, while lower SE was obtained ranging between 0.006–0.034 in the multi-trait Bayesian analysis (Table 4). A significantly reduced error can be explained by the normalization of skewed and biased estimates in Bayesian approach for all considered traits in Tharparkar cattle.

Heritability of AFC estimated as 0.52±0.07 was found in close agreement with a previous study, while for FSP (h^2^ = 0.12±0.17) it did not agree in the Tharparkar cattle [38]. Similar moderate heritability estimates for first lactation traits were observed by Choudhary et al [38] and Taneja et al [41] in Tharparkar cattle. Heritability estimates of LTMY, PL and HL was previously reported as 0.50±0.39, 0.73±0.32, and 0.80±0.44, respectively [38], which was higher compared to the present mean posterior estimates, while the SE of these estimates was also higher from MCE. Estimates of (co)variances for all the traits in this study could be used in future as prior values along with the temporal data distribution to estimate more accurate and precise posterior values, respectively.

### Genetic and phenotypic correlations

The moderate genetic correlation of AFC and FSP indicated more influence at genetic level as compared to the environmental factors, as the higher values (in days) of both these reproduction traits are undesirable for economic production of any herd. Indirect selection was preferable from AFC in dairy cattle for linear type traits [42,43]. The reproductive traits are economically very important as the AFC and SP have reasonable correlation with various conformation, fertility, and lifetime traits of dairy animals [44–46]. For long term selection purpose multi-trait analysis are more useful through the early and indirect selection of all the correlated traits (Tables 5 and 6).

Genetic and phenotypic correlations between various traits estimated through the LSML approach in the Tharparkar cattle didn’t show any definite patterns (Table 5). Negative genetic and phenotypic correlation was noticed for the AFC-FLMY, AFC-FLFY, AFC-FLSNFY, AFC-LTMY, FSP-LTMY, FSP-PL, LTMY-FLSNFY, PL-FSP, PL-FLMY, PL-FLFY and PL-FLSNFY, while these correlations were positive for the remaining traits in the present study. Most of these correlations were inconsistent with the production potential of herd, viz. negative correlation of PL with milk composition traits. Therefore, MTGSAM approach was utilized for more efficient and reliable estimation of various genetic and phenotypic correlations among these traits in the Tharparkar cattle (Table 6).

Our result indicated that the LTMY did not converge to the desired distribution in the multi-trait Bayesian analysis due to its very higher variability. High variability of the LTMY trait was not suitable for the normalization and to achieve positive definite matrix with considered economic traits for parameter estimation. First lactation production status reflected PL in the Tharparkar cattle to some extent; however, the effect of other factors and their interaction effects could also play significant role in the subsequent lactations in different parities. This indicated that the combined study of first lactation traits with the lifetime traits were required to define the genetic potential of the Tharparkar herd. Indirect selection could result in the improvement of these traits based on the correlation components. Our result revealed little scope of improvement in the lifetime traits due to their low phenotypic and genetic correlations (FLMY-PL/HL, FLFY-PL/HL, and FLSNFY-PL/HL); high to moderate scope of improvement of production with the reproduction traits due to medium correlations among these traits (FLMY-AFC/FSP, FLFY-AFC/FSP, and FLSNFY-AFC/FSP); while higher scope genetic improvement in the production and lifetime traits due to high and positive genetic and phenotypic correlations among these traits (FLMY-FLSNFY, FLMY-FLFY and FLFY-FLSNFY, PL-HL). Our study also revealed high correlations for FLFY and FLSNFY by trivariate analysis, indicating short term improvement by indirect selection, as these traits appeared simultaneously in animal’s life span and affected by common non-genetic and genetic factors. Better opportunity for improvement can be obtained by indirect selection with the PL and HL as genetic and phenotypic correlations were higher.

All the reproduction, production and the lifetime traits were positively correlated, except the AFC and FSP which were negatively correlated with the PL and HL which was favourable. AFC and FSP were found as most promising traits for the indirect selection with a long-term goal based on their favourable genetic and phenotypic correlations in the Tharparkar cattle. High genetic improvement could be expected from the indirect selection of these traits in the ongoing breeding program for Tharparkar herd. The negative genetic correlations indicated that the lower estimates of the AFC and FSP were in the desirable directions for the economic benefit of the herd by ensuring higher PL and HL. Our results pointed towards the scope for long term improvement in the production and lifetime traits based on indirect selection of the AFC due to their favourable genetic and phenotypic correlations in the Tharparkar cattle.

### Effectiveness of sire evaluation

The Tharparkar sires were evaluated based on the EBVs generated by both the approaches, using the rank correlation which was intended for calculating the t-values (Table 7). These calculated t-values were used for comparison with tabulated t-values at 5% and 1% level of significance at appropriate degrees of freedom. The ranking of most of the sires was the same for the highest breeding value based on the first lactation traits in the Tharparkar cattle. However, overall ranking for all the sires were not the the same by both methods. Under these circumstances, it was necessary to compare the ranking of sires. Due to this difference in ranking of sires, the significance of rank correlation was judged by t-test. Non-significant difference was noticed in ranking of the sires by these methods due to a smaller number of sires with this sampling structure (Table 7).

## CONCLUSION

The goals of decision-making in subsidized farms are to lower the costs of rearing and save time, which can be achieved by an accurate and precise estimate of genetic parameters with a simple analytic process. Considering such objectives, the results of the present study were important to increase profitability and genetic potential in dairy cattle reproduction, and ultimately lifetime productivity. Distributions provided by the Bayesian analysis have great flexibility in cross checking, visualization, and automation of statistical analyses from marginal posterior distributions which was not possible with the conventional methods of analysis. BLUPF90 is a user-friendly tool to compute efficient solutions for genetic parameters even with a small dataset. The moderate to higher value of heritability estimate for the considered traits indicated sufficient genetic base for efficient selection in the Tharparkar cattle. Selection of productive animals based on milk constituents (FLFY and FLSNFY) is recommended for indirect selection with short-term goals in Tharparkar. Correlations studies enhance the scope of indirect selection at an early age by AFC and FSP for production potential in long-term breeding programs of the Tharparkar herd. Our results revealed that the AFC had a favourable negative genetic correlation with the production and lifetime traits, which indicated that the AFC will be an important trait for indirect selection of these production traits, since a reduction in AFC will be beneficial for improving first lactation and lifetime traits in the Tharparkar cattle. Distinct ranking and efficient evaluation of the Tharparkar sires based on the Bayesian approach, compared to that of the frequentist LSML technique, suggested the multi-trait Bayesian analysis to be the method of choice for the reliable genetic assessment of the Tharparkar cattle.

## ETHICAL STATEMENT

No animal were harmed and/or not fed anything for experimental purpose other than regular daily allowances, during the presented research work as previously collected information for considered traits were utilized with permission from the livestock record unit, ICAR-NDRI, Karnal, Haryana, India. All animal welfare rights were reserved while conducting this study.

## Figures and Tables

**Table 1:** Descriptive statistics

Parameters	Mean±SE	CV	SD	Minimum	Maximum
Reproduction traits					
AFC	1,227.73±24.88	14.62	179.45	879	1,554
FSP	148.37±13.06	63.50	94.21	41	374
Production traits					
FLMY	1,765.44±108.89	39.49	697.26	506	3,149.1
FLFY	2,701.79±63.93	15.15	409.36	2,029.83	3,579.81
FLSNFY	1,482.38±42.01	18.15	269.02	952.05	2,012.39
Life time traits					
LTMY	5,374.76±455.01	58.04	3119.39	701	13,433.6
PL	1,292.72±93.34	49.50	639.90	492	3,010
HL	2,555.11±95.63	25.66	655.63	1,650	4,233

SE, standard error; CV, coefficient of variation (%); SD, standard deviation; AFC, age at first calving; FSP, first service period; FLMY, first lactation milk yield; FLFY, first lactation fat yield; FLSNFY, first lactation solid-not fat yield; LTMY, lifetime milk yield; PL, productive life; HL, herd life; N, no. of records.

**Table 2:** Least-squares means

Traits	LSM±SE	Effect of period	Effect of season	Effect of covariate	Sire
AFC (d)	1,093.74±31.83	^*^	^***^	^*^	^*^
FSP (d)	152.49±17.25	^*^	^*^	^*^	^*^
FLMY (kg)	1,777.95±211.60	^*^	^*^	^*^	^*^
FLSNFY (kg)	2,962.03±186.61	^*^	^*^	^*^	^*^
FLFY (kg)	1,633.76±105.86	^***^	^*^	^*^	^*^
LTMY (kg)	7,875.39±845.09	^**^	^*^	^*^	^**^
PL (d)	5,373.64±186.15	^*^	^*^	^*^	^*^
HL (d)	6,665.89±189.19	^*^	^*^	^*^	^*^

LSM, least squares means; SE, standard error; AFC, age at first calving; FSP, first service period; FLMY, first lactation milk yield; FLSNFY, first lactation solid-not fat yield; FLFY, first lactation fat yield; LTMY, lifetime milk yield; PL, productive life; HL, herd life.

* Non-significant;

** p<0.05,

*** p<0.01.

**Table 3:** Descriptive statistics for variance components and heritability estimates by MTGSAM approach for reproduction, production and lifetime traits

Parameters		Mean	Mode	Median	SD	HPD (95%)		Effective size	MCE^SD^	Geweke diagnostic

						Lower	Upper			
Reproduction traits										
AFC	σ^2^_g_	21,689	19,541	20,490	8,667.5	7,404.0	40,300	145.8	717.70	0.16
	σ^2^_p_	35,734	29,424	34,270	9,305.5	2,0067	53,420	233.1	609.28	0.06
	σ^2^_e_	14,045	12,486	12,710	7,566.5	2,056.0	28,170	149.5	618.54	−0.10
	h^2^	0.60712	0.58322	0.62218	0.17126	0.29012	0.93540	101.2	0.017	0.17
FSP	σ^2^_g_	10,175	4,769.9	7,830.0	8,456.1	426.70	25,580	50.3	1,192.2	−0.04
	σ^2^_p_	17,265	9,600.6	14,134	10,856	4548.0	38,220	56.2	1,448.4	0.03
	σ^2^_e_	7,089.9	4,144.7	5,670.0	4,975.8	849.60	17,440	149.8	406.44	0.15
	h^2^	0.56587	0.61682	0.57724	0.18919	0.21388	0.91627	61.6	0.024	−0.30
Production traits										
FLMY	σ^2^_g_	2,589,900	1,022,200	2,100,000	1,848,700	345,500	6,414,000	35.2	311,430	−0.27
	σ^2^_p_	4,453,900	2,220,200	3,801,000	2,617,600	1,088,100	9,651,000	41.0	408,740	−0.24
	σ^2^_e_	1,864,000	866,080	1,561,000	1,228,900	231,400	4308,000	79.2	138,050	−0.10
	h^2^	0.56706	0.57248	0.57226	0.15550	0.26959	0.85375	61.5	0.0198	−0.19
FLSNFY	σ^2^_g_	811,450	489,010	726,500	421,260	150,300	1,650,000	57.1	55,734	−0.24
	σ^2^_p_	1,345,100	877,040	1,220,000	577,850	455,600	2,456,300	79.8	64,678	−0.25
	σ^2^_e_	533,640	373,470	469,300	288,580	116,700	1,134,000	206.4	20,083	−0.15
	h^2^	0.59619	0.63443	0.60330	0.14069	0.33115	0.86198	116.0	0.01306	−0.13
FLFY	σ^2^_g_	2,831,800	1,789,600	251,9000	1,495,000	650,700	5,689,000	102.1	147,910	−0.15
	σ^2^_p_	4,702,600	3,531,400	426,0000	2,125,000	1,604,800	8,692,000	101.2	211,240	−0.16
	σ^2^_e_	1,870,800	1,305,400	1,635,000	1,067,800	401,100	3,989,000	185.2	78,457	−0.10
	h^2^	0.59850	0.61924	0.60614	0.13709	0.33256	0.84968	570.5	0.00574	−0.09
Life time traits										
LTMY	σ^2^_g_	2,956,500	2,077,100	2,554,000	2,201,100	152,300	6,249,000	208.2	152,470	−0.02
	σ^2^_p_	12,187,000	9,603,500	11,499,000	3,791,300	6,478,000	19,506,000	463.9	175,930	0.13
	σ^2^_e_	9,230,900	8,297,200	8,699,000	3,157,800	3,775,000	15,550,000	782.5	112,830	0.17
	h^2^	0.2406	0.25031	0.2262	0.1206	0.0366	0.4703	191.5	0.0087	−0.18
PL	σ^2^_g_	276,470	240,740	256,300	144,640	34,270	552,800	52.1	20,038	0.08
	σ^2^_p_	654,990	530,860	621,600	190,260	349,800	1,056,300	229.9	12,545	0.14
	σ^2^_e_	378,520	277,460	358,700	158,040	105,200	684,200	106.6	15,300	0.09
	h^2^	0.42039	0.44293	0.41398	0.17009	0.09959	0.72977	47.7	0.02463	−0.01
HL	σ^2^_g_	247,420	212,570	223,500	118,870	74,730	466,300	60.3	15,297	−0.02
	σ^2^_p_	660,240	546,790	626,100	191,180	343,500	1,025,700	118.8	17,535	0.26
	σ^2^_e_	412,820	327,950	389,900	174,190	127,400	788,300	28.1	32,844	0.30
	h^2^	0.38241	0.34443	0.36557	0.15157	0.12812	0.67899	19.5	0.0344	−0.21

MTGSAM, multi-trait Bayesian-Gibbs sampler approach; SD, standard deviation; HPD(95%), higher posterior density 95%;; MCE^SD^, Monte Carlo error for SD; σ^2^_g_, additive genetic variance; σ^2^_p_, total phenotypic variance; σ^2^_e_, residual variance; h^2^, heritability; AFC, age at first calving; FSP, first service period; FLMY, first lactation milk yield; FLFY, first lactation fat yield; FLSNFY, first lactation solid-not fat yield; LTMY, lifetime milk yield; PL, productive life; HL, herd life.

**Table 4:** Comparative summary of heritability estimate

Traits	LSML	Bayesian
Reproduction traits		
AFC	0.20±0.441	0.61±0.017
FSP	0.23±0.568	0.57±0.024
Production traits		
FLMY	0.34±0.605	0.57±0.020
FLSNFY	0.31±0.697	0.60±0.013
FLFY	0.24±0.679	0.60 ±0.006
Lifetime traits		
LTMY	0.25±0.731	0.24±0.009
PL	0.49±0.709	0.42±0.025
HL	0.48±0.705	0.38±0.034

LSML, least squares maximum likelihood; AFC, age at first calving; FSP, first service period; FLMY, first lactation milk yield; FLSNFY, first lactation solid-not fat yield; FLFY, first lactation fat yield; LTMY, lifetime milk yield; PL, productive life; HL, herd life.

**Table 5:** The posterior mean of genetic (above diagonal), phenotypic (below the diagonal) correlations and heritability estimates (diagonal) for traits using LSML approach in Tharparkar

Traits	AFC	FSP	FLMY	FLFY	FLSNFY	LTMY	PL	HL
AFC	0.20±0.441	0.1059	−0.0295	−0.1237	−0.0971	−0.0566	0.0330	NA
FSP	0.1042	0.23±0.568	0.1412	0.3284	0.2804	−0.3640	−0.3376	NA
FLMY	−0.0750	0.1928	0.34±0.605	0.6710	0.6591	0.1423	−0.0265	NA
FLFY	−0.1765	0.2915	0.6397	0.24±0.679	0.9622	−0.0134	−0.2215	NA
FLSNFY	−0.1597	0.2956	0.6580	0.9486	0.31±0.697	−0.0037	−0.2009	NA
LTMY	−0.0466	−0.3821	0.6580	0.0202	−0.0443	0.25±0.731	NA	NA
PL	0.0565	−0.3485	−0.0159	−0.1552	−0.2224	NA	0.49±0.709	NA
HL	NA	NA	NA	NA	NA	NA	NA	0.48±0.705

LSML, least squares maximum likelihood; NA, not applicable; AFC, age at first calving; FSP, first service period; FLMY, first lactation milk yield; FLFY, first lactation fat yield; FLSNFY, first lactation solid-not fat yield; LTMY, lifetime milk yield; PL, productive life; HL, herd life.

**Table 6:** The posterior mean of genetic (above diagonal), phenotypic (below the diagonal) correlations and heritability estimates (diagonal) with standard error using MTGSAM approach in Tharparkar

Traits	AFC	FSP	FLMY	FLFY	FLSNFY	PL	HL
AFC	0.61±0.017	0.32±0.050	0.68±0.018	0.80±0.008	0.85±0.007	−0.59±0.19	−0.59±0.24
FSP	0.46±0.037	0.57±0.024	0.59±0.056	0.65±0.074	0.63±0.075	−0.38±0.101	−0.34±0.076
FLMY	0.77±0.013	0.54±0.049	0.57±0.020	0.92±0.007	0.93±0.007	0.13±0.133	0.23±0.117
FLFY	0.85±0.005	0.64±0.036	0.92±0.006	0.60±0.013	0.99±0.0005	0.33±0.093	0.25±0.120
FLSNFY	0.87±0.004	0.64±0.037	0.93±0.006	0.99±0.0003	0.60±0.006	0.35±0.091	0.26±0.121
PL	−0.33±0.029	−0.16±0.067	0.19±0.054	0.23±0.0691	0.24±0.0553	0.42±0.025	0.95±0.0032
HL	−0.21±0.029	−0.04±0.041	0.25±0.047	0.23±0.0525	0.23±0.0488	0.96±0.0032	0.38±0.034

MTGSAM, multi-trait Gibbs sampling animal model; AFC, age at first calving; FSP, first service period; FLMY, first lactation milk yield; FLFY, first lactation fat yield; FLSNFY, first lactation solid-not fat yield; PL, productive life; HL, herd life.

**Table 7:** Effectiveness of sire evaluation

Trait	Rank correlation	t-calculated	df	Interpretation
FLMY	0.048	0.12	7	Non-significant
FLSNFY	0.39	1.04		
FLFY	0.43	1.17		

FLMY, first lactation milk yield; FLSNFY, first lactation solid-not fat yield; FLFY, first lactation fat yield.

## Data Availability

The data are available upon reasonable request from the corresponding author.
